# Supplementary material for: A Survey of Paediatric Rapid Sequence Induction in a Department of Anaesthesia
Source: Children (Basel). 2022 Sep 19;9(9):1416. doi: 10.3390/children9091416 (PMC9497683; doi:10.3390/children9091416)
Supplement: Supplementary file 1 [file children-09-01416-s001.zip › children-1877775-supplementary.pdf]

# A survey of rapid sequence induction (RSI) and intubation practice in paediatric patients

Tick the appropriate block

1. What grade of anaesthetist are you?

| | |
|---|---|
| Medical officer | |
| Junior registrar (<2yrs) | |
| Senior registrar (>2yrs) | |
| Principal medical officer | |
| Consultant < 5 years | |
| Consultant 5 – 10 years | |
| Consultant >10 years | |

2. For registrars only – How long ago did you complete your paediatric training block?

| | |
|---|---|
| Have not yet done the block | |
| Currently doing the block | |
| 0 - 12 months ago | |
| 1 – 2 years ago | |
| > 2 years ago | |

3. For consultants only – How often do you anaesthetise children?

| | |
|---|---|
| At least one list per week | |
| At least one list per month | |
| Less than one list per month | |
| Never | |

4. For which of the following cases would you use a RSI technique?
   (You can choose more than one option)

| | |
|---|---|
| Any emergency case | |
| A non-fasted patient | |
| An upper GIT obstruction | |
| A lower GIT obstruction | |
| Appendicitis with localized pain and soft abdomen | |
| Appendicitis with distended abdomen and generalized peritonitis | |
| A patient with bulbar palsy | |
| Insulin dependent diabetes mellitus | |
| Renal failure | |

5. In your opinion, what are the **2** most important components of a *controlled* RSI? (Choose 2)

| | |
|---|---|
| Attempting laryngoscopy and intubation immediately after induction | |
| Maintaining oxygenation using appropriate bag-mask ventilation | |
| Waiting for the 'magical minute' before intubating the patient | |
| The use of cricoid pressure | |
| Ensuring adequate depth of anaesthesia and paralysis before intubation | |

**During a RSI for paediatric patients:**

Key:

| | |
|---|---|
| Neonate | < 28 days old |
| Infant | 1 month-1 year |
| Child | 1-12 years |

1. Which is your preferred method of pre-oxygenation for

**(A) a neonate:**

| | | | |
|---|---|---|---|
| Normal tidal breathing<br>If so, for how long? …………….. | | Do not attempt pre-oxygenation | |

**(B) an infant:**

| | | | |
|---|---|---|---|
| Normal tidal breathing<br>If so, for how long? …………….. | | Do not attempt pre-oxygenation | |

**(C) a child**

| | | | | | |
|---|---|---|---|---|---|
| Normal tidal breathing<br>If so, for how long? ………. | | Vital capacity breaths. If so, how many?......... | | Do not attempt pre-oxygenation | |

2. Which induction agent do you use in a haemodynamically stable patient for

**(A) a neonate**

| | | | | | | | | | |
|---|---|---|---|---|---|---|---|---|---|
| Propofol | | Ketamine | | Etomidate | | Sevoflurane | | Other (specify) | |

**(B) an infant**

| | | | | | | | | | |
|---|---|---|---|---|---|---|---|---|---|
| Propofol | | Ketamine | | Etomidate | | Sevoflurane | | Other (specify) | |

**(C) a child**

| | | | | | | | | | |
|---|---|---|---|---|---|---|---|---|---|
| Propofol | | Ketamine | | Etomidate | | Sevoflurane | | Other (specify) | |

3. Which paralysing agent do you use during induction for

**(A) a neonate**

| | | | | | | | | | |
|---|---|---|---|---|---|---|---|---|---|
| Suxamethonium | | Rocuronium | | Cis-/atracurium | | None | | Other (specify) | |

**(B) an infant**

| | | | | | | | | | |
|---|---|---|---|---|---|---|---|---|---|
| Suxamethonium | | Rocuronium | | Cis-/atracurium | | None | | Other (specify) | |

**(C) a child**

| | | | | | | | | | |
|---|---|---|---|---|---|---|---|---|---|
| Suxamethonium | | Rocuronium | | Cis-/atracurium | | None | | Other (specify) | |

4. Do you use cricoid pressure in

**(A) a neonate**

| | | | |
|---|---|---|---|
| YES | | NO | |

**(B) an infant**

| | | | |
|---|---|---|---|
| YES | | NO | |

**(C) a child**

| | | | |
|---|---|---|---|
| YES | | NO | |

5. Do you use bag-mask-ventilation during a RSI in

**(A) a neonate**

| | | | |
|---|---|---|---|
| YES | | NO | |

**(B) an infant**

| | | | |
|---|---|---|---|
| YES | | NO | |

**(C) a child**

| | | | |
|---|---|---|---|
| YES | | NO | |

If so, what is your maximum inspiratory pressure used while bag-mask-ventilating the patient?

| | |
|---|---|
| <12 cmH20 | |
| 12 – 16 cmH20 | |
| >16 cmH20 | |

6. Do you use a nerve stimulator to monitor adequate paralysis prior to intubation?

| | | | |
|---|---|---|---|
| YES | | NO | |

7. Do you use ultrasound to assess the presence of gastric content prior to a RSI?

| | | | |
|---|---|---|---|
| YES | | NO | |

8. Do you routinely assess clinical parameters of volume-status prior to performing a RSI for a paediatric patient?

| | | | |
|---|---|---|---|
| YES | | NO | |

9. Do you use a cuffed or uncuffed endotracheal tube for a paediatric patient at risk of pulmonary aspiration in:

**(A) a neonate**

| | | | |
|---|---|---|---|
| cuffed | | uncuffed | |

**(B) an infant**

| | | | |
|---|---|---|---|
| cuffed | | uncuffed | |

**(C) a child**

| | | | |
|---|---|---|---|
| cuffed | | uncuffed | |

10. Would you find it useful to have guidelines which direct you on the conduct of RSI for paediatric patients?

| | | | |
|---|---|---|---|
| YES | | NO | |

**Clinical Scenarios:**

You are asked to anaesthetise the patient in the following scenarios:

Please describe the technique you would most commonly use to establish anaesthesia by indicating on the table, the components you would use and the order you would use them:
**(1= first action, 2= second action, and so forth. You do not have to use all components)**

1. **A 3-week-old, otherwise healthy baby, for a pyloromyotomy. He has been fully resuscitated with IV fluids but the cannula came out during transfer to OT. He has a nasogastric tube in-situ that appears to be draining well.**

| **Components** | **Use (Circle)** | **Sequence order (Circle)** |
|---|---|---|
| Site intravenous access | Y / N | N/A 1 2 3 4 5 6 7 8 |
| Insert/suction naso/orogastric tube | Y / N | N/A 1 2 3 4 5 6 7 8 |
| Pre-oxygenation | Y / N | N/A 1 2 3 4 5 6 7 8 |
| Induction | Intravenous/ Inhalational | N/A 1 2 3 4 5 6 7 8 |
| Cricoid pressure | Y / N | N/A 1 2 3 4 5 6 7 8 |
| Appropriate Bag-mask-ventilation | Y / N | N/A 1 2 3 4 5 6 7 8 |
| Muscle relaxant<br>• Suxamethonium (Sux)<br>• Rocuronium (Roc)<br>• Cis/-Atracurium (Cis-Atr)<br>• None (Ø) | <u>Circle drug of choice</u><br>Sux / Roc / Cis-Atr/ Ø | N/A 1 2 3 4 5 6 7 8 |
| Definitive airway control<br>• Laryngeal mask airway (LMA)<br>• Cuffed endotracheal tube (cETT)<br>• Uncuffed endotracheal tube (uETT) | LMA / cETT / uETT | N/A 1 2 3 4 5 6 7 8 |

**2. A 4-year-old, previously healthy child who has suspected small bowel obstruction. He has been unwell for 48hrs, with episodes of vomiting and a tender, distended abdomen. He has an IV line running and has been resuscitated. There is no naso/orogastric tube in place.**

| **Components** | **Use (Circle)** | **Sequence order (Circle)** |
|---|---|---|
| Site intravenous access | Y / N | N/A 1 2 3 4 5 6 7 8 |
| Insert/suction naso/orogastric tube | Y / N | N/A 1 2 3 4 5 6 7 8 |
| Pre-oxygenation | Y / N | N/A 1 2 3 4 5 6 7 8 |
| Induction | Intravenous/<br>Inhalational | N/A 1 2 3 4 5 6 7 8 |
| Cricoid pressure | Y / N | N/A 1 2 3 4 5 6 7 8 |
| Appropriate Bag-mask-ventilation | Y / N | N/A 1 2 3 4 5 6 7 8 |
| Muscle relaxant<br><br>• Suxamethonium (Sux)<br>• Rocuronium (Roc)<br>• Cis/-Atracurium (Cis-Atr)<br>• None (Ø) | Circle drug of choice<br><br>Sux / Roc / Cis-Atr/ Ø | N/A 1 2 3 4 5 6 7 8 |
| Definitive airway control<br><br>• Laryngeal mask airway (LMA)<br>• Cuffed endotracheal tube (cETT)<br>• Uncuffed endotracheal tube (uETT) | LMA / cETT / uETT | N/A 1 2 3 4 5 6 7 8 |

**3. A 6-year-old, otherwise healthy child who has a painful forearm fracture requiring manipulation. He ate 2hrs prior to the injury and has now been starved for 6hrs since the injury. He has received opioids in the pre-operative period.**

| **Components** | **Use (Circle)** | **Sequence order (Circle)** |
|---|---|---|
| Site intravenous access | Y / N | N/A 1 2 3 4 5 6 7 8 |
| Insert/suction naso/orogastric tube | Y / N | N/A 1 2 3 4 5 6 7 8 |
| Pre-oxygenation | Y / N | N/A 1 2 3 4 5 6 7 8 |
| Induction | Intravenous/<br>Inhalational | N/A 1 2 3 4 5 6 7 8 |
| Cricoid pressure | Y / N | N/A 1 2 3 4 5 6 7 8 |
| Appropriate Bag-mask-ventilation | Y / N | N/A 1 2 3 4 5 6 7 8 |
| Muscle relaxant<br><br>• Suxamethonium (Sux)<br>• Rocuronium (Roc)<br>• Cis/-Atracurium (Cis-Atr)<br>• None (Ø) | Circle drug of choice<br><br>Sux / Roc / Cis-Atr/ Ø | N/A 1 2 3 4 5 6 7 8 |
| Definitive airway control<br><br>• Laryngeal mask airway (LMA)<br>• Cuffed endotracheal tube (cETT)<br>• Uncuffed endotracheal tube (uETT) | LMA / cETT / uETT | N/A 1 2 3 4 5 6 7 8 |

PTO

4. **A 7-year-old with renal failure and ascites who has been booked for Tenckhoff catheter insertion, in order to commence dialysis. It is an elective procedure and the child has been starved:**

| **Components** | **Use (Circle)** | **Sequence order (Circle)** |
|---|---|---|
| Site intravenous access | Y / N | N/A 1 2 3 4 5 6 7 8 |
| Insert/suction naso/orogastric tube | Y / N | N/A 1 2 3 4 5 6 7 8 |
| Pre-oxygenation | Y / N | N/A 1 2 3 4 5 6 7 8 |
| Induction | Intravenous/<br>Inhalational | N/A 1 2 3 4 5 6 7 8 |
| Cricoid pressure | Y / N | N/A 1 2 3 4 5 6 7 8 |
| Appropriate Bag-mask-ventilation | Y / N | N/A 1 2 3 4 5 6 7 8 |
| Muscle relaxant<br>• Suxamethonium (Sux)<br>• Rocuronium (Roc)<br>• Cis/-Atracurium (Cis-Atr)<br>• None (Ø) | <u>Circle drug of choice</u><br>Sux / Roc / Cis-Atr/ Ø | N/A 1 2 3 4 5 6 7 8 |
| Definitive airway control<br>• Laryngeal mask airway (LMA)<br>• Cuffed endotracheal tube (cETT)<br>• Uncuffed endotracheal tube (uETT) | LMA / cETT / uETT | N/A 1 2 3 4 5 6 7 8 |
